# Supplementary material for: Topaz-Denoise: general deep denoising models for cryoEM and cryoET
Source: Nat Commun. 2020 Oct 15;11:5208. doi: 10.1038/s41467-020-18952-1 (PMC7567117; doi:10.1038/s41467-020-18952-1)
Supplement: Supplementary file 1 — Supplementary Information [file 41467_2020_18952_MOESM1_ESM.pdf]

Supplementary Information

Topaz-Denoise: general deep denoising models for cryoEM and cryoET

Tristan Bepler *et al*.

Training Dataset	Model architecture	Objective	EMPIAR datasets			NYSBC K2 datasets				NYSBC Falcon III datasets			Overall
			10261	10005	10025	Protocadherin	18sep08d	19jan04d	19may10e	18aug17l	18sep06d	18sep19l	
Small	Affine	L1	5.49	1.29	0.72	4.83	4.51	8.87	12.02	10.65	6.90	9.15	6.44
		L2	5.28	1.04	0.40	4.64	4.33	8.64	11.88	10.43	6.89	9.02	6.25
	FCNN	L1	5.45	1.25	-0.05	5.37	5.85	8.78	12.77	12.02	7.90	10.89	7.02
		L2	5.17	0.94	-0.59	4.82	5.67	8.62	12.31	11.30	7.76	10.29	6.63
	U-Net (small)	L1	5.99	0.65	0.12	5.89	**7.11**	9.71	14.17	12.05	**8.25**	10.93	7.49
		L2	5.32	0.49	0.27	5.55	6.85	**9.74**	**14.26**	11.23	7.49	10.36	7.16
	U-Net	L1	5.24	0.79	2.51	5.64	5.38	9.38	13.53	9.91	6.49	9.59	6.85
		L2	5.72	0.86	**3.01**	5.41	5.22	8.64	13.06	9.36	6.61	9.16	6.70
Large	Affine	L1	4.30	-0.24	-1.09	3.76	3.36	7.23	11.34	9.32	6.56	8.45	5.30
		L2	4.26	-0.27	-1.15	3.74	3.33	7.20	11.32	9.32	6.57	8.46	5.28
	FCNN	L1	4.69	-0.74	-1.57	3.95	5.25	7.36	11.93	10.26	8.04	9.72	5.89
		L2	3.78	-0.47	-1.57	3.76	4.79	6.81	11.21	10.15	7.34	9.62	5.54
	U-Net (small)	L1	6.47	0.51	-1.00	5.18	4.82	7.04	12.29	13.62	8.04	10.83	6.78
		L2	6.00	0.84	-0.46	5.19	5.20	7.90	12.34	12.36	7.20	10.38	6.69
	U-Net	L1	6.95	1.88	0.83	**6.33**	6.14	9.35	13.87	13.11	7.35	12.76	7.86
		L2	**7.17**	1.72	1.07	5.94	6.06	8.43	13.07	**15.17**	7.37	**13.24**	**7.92**
Low-pass		4	-5.28	-11.17	-11.92	-5.28	-6.08	-2.97	3.44	-1.04	-1.02	-0.29	-4.16
		8	0.30	-5.52	-5.89	-0.23	-0.89	2.03	7.84	4.30	2.87	4.57	0.94
		16	5.19	-0.12	-0.40	4.22	3.53	6.87	9.99	9.04	6.95	8.71	5.40
		32	3.92	**1.89**	0.08	1.65	1.63	8.25	10.13	8.58	1.90	7.92	4.59
		64	2.34	-2.22	-0.83	-3.25	5.60	5.18	11.12	10.23	6.58	-0.02	3.47
Raw			-17.14	-20.13	-24.15	-14.47	-15.40	-11.73	-5.44	-6.33	-3.64	-5.63	-12.41

Supplementary Table 1 | SNR comparison for model architectures, loss functions, and training datasets for the 2D denoising models. Comparison of denoising methods based on estimated SNR (in dB, larger is better). SNR was estimated from 20 paired signal and background regions selected for each dataset. In each column, the best performing model is highlighted. We report denoising results on aligned and dose weighted micrographs for the NYSBC K2 and Falcon III datasets.All datasets were collected in electron counting modes, except for 18sep06d which was collected using Falcon III integrating mode. The U-net denoising model trained on the “Large” dataset with L2 loss performs best on average. For the low-pass filter baselines, the amount of filtering is reported in the “Objective” column. The SNR of the raw micrographs is reported in the last row.

Method	19aug10b: Yeast lamella	19jan20b: Cadherins on liposomes	19jul11a: Yeast lamella	19oct06a: Cellular lamella
Raw (bin2)	-11.12	-14.33	-9.27	-12.91
Low-pass (2x)	-9.04	-14.08	-6.71	-11.83
Low-pass (4x)	-8.44	-13.31	-5.03	-11.12
Low-pass (8x)	-12.34	-13.51	-8.63	-14.58
Topaz (self-trained)	-7.27	-11.55	-4.73	-9.78
Topaz (Unet-3d-10a)	-7.55	-11.66	-4.79	-10.00

Supplementary Table 2 | SNR comparison for model architectures and training datasets for the 3D denoising models. Comparison of tomogram denoising methods by SNR (in dB). For each dataset, SNR was calculated following the independent signals method. Briefly, tomograms were split into even/odd frame tomograms, denoising methods were applied to the even frame tomograms and odd frame tomograms independently. Then, denoised even frame tomograms were compared to the raw odd frame tomograms to calculate SNR and vice versa for the denoised odd and raw even frame tomograms. These values were averaged together to give the reported SNRs. The Topaz U-net models trained on each tomogram (self-trained) achieves the best SNR in each case, but is only marginally better than the general pre-trained tomogram denoising model (Unet-3d-10a). Both models improve over low-pass filtering on all four examples.

Dataset Name	Number of Micrographs	Description
NYSBC zero defocus	671	NYSBC ladder dataset collected at zero defocus; K2 counting
NYSBC ice images	900	NYSBC micrographs taken of vitreous ice; K2 counting
EMPIAR-10025[49]	196	T20S proteasome; K2 super-resolution
EMPIAR-10005[24]	499	TRPV1; K2 counting
EMPIAR-10210[50]	170	mouse MDA5-dsRNA filaments; Falcon III counting
EMPIAR-10243[51]	142	heparin-induced 2N4R tau filaments; Falcon III counting
EMPIAR-10244[52]	642	RNA polymerase II transcribing a nucleosome; K2 super-resolution
EMPIAR-10248[53]	971	Apoferritin by CRYOARM300 with cold-FEG; K2 counting
EMPIAR-10249[5]	596	Horse liver alcohol dehydrogenase movies obtained using Talos Arctica operating at 200 kV equipped with a K2
EMPIAR-10250[5]	181	Human methemoglobin movies obtained using Talos Arctica operating at 200 kV equipped with a K2
EMPIAR-10252[5]	153	Catalytic subunit of protein kinase A bound to ATP, manganese, and IP20 movies obtained using Talos Arctica operating at 200 kV equipped with a K2
EMPIAR-10257[54]	295	NDH the complex I-like molecule of photosynthesis; K2 super-resolution
EMPIAR-10258[55]	199	LRRC8A-DCPIB in MSP1E3D1 nanodiscs; K2 super-resolution
EMPIAR-10259[55]	198	apo-LRRC8A in MSP2N2 nanodiscs; K2 super-resolution
EMPIAR-10261[48]	1461	ProTx2-bound Nav1.7 VSD2-NavAb chimeric channel; K2 counting
EMPIAR-10031[56]	512	MAVS CARD C1 filaments, Falcon II counting
EMPIAR-10061[57]	397	beta-galactosidase in complex with a cell-permeant inhibitor; K2 super-resolution
EMPIAR-10028[58]	600	Plasmodium falciparum 80S ribosome bound to the anti-protozoan drug emetine; Falcon II counting
Small	1929	Contains micrographs from datasets: EMPIAR-10005, -10025, -10061, -10244, -10249, -10250, -10252, -10257, -10258, and -10261
Large	3439	Contains micrographs from all individual datasets

Supplementary Table 3 | Training datasets for the 2D denoising models. The individual datasets with the number of micrographs from each and brief descriptions are provided in the first block. The second block describes the camera/imaging mode specific datasets. These are composed of all micrographs from subsets of the individual datasets. The last block describes the two general datasets. The “Small” dataset is composed of micrographs from a subset of the individual datasets that we found to give best performing models by eye. The “Large” dataset contains micrographs from all individual datasets. For the “Small” and “Large” datasets, individual datasets with more than 200 micrographs were subsampled to only include 200 images. This serves to approximately balance the contributions of each contained dataset.

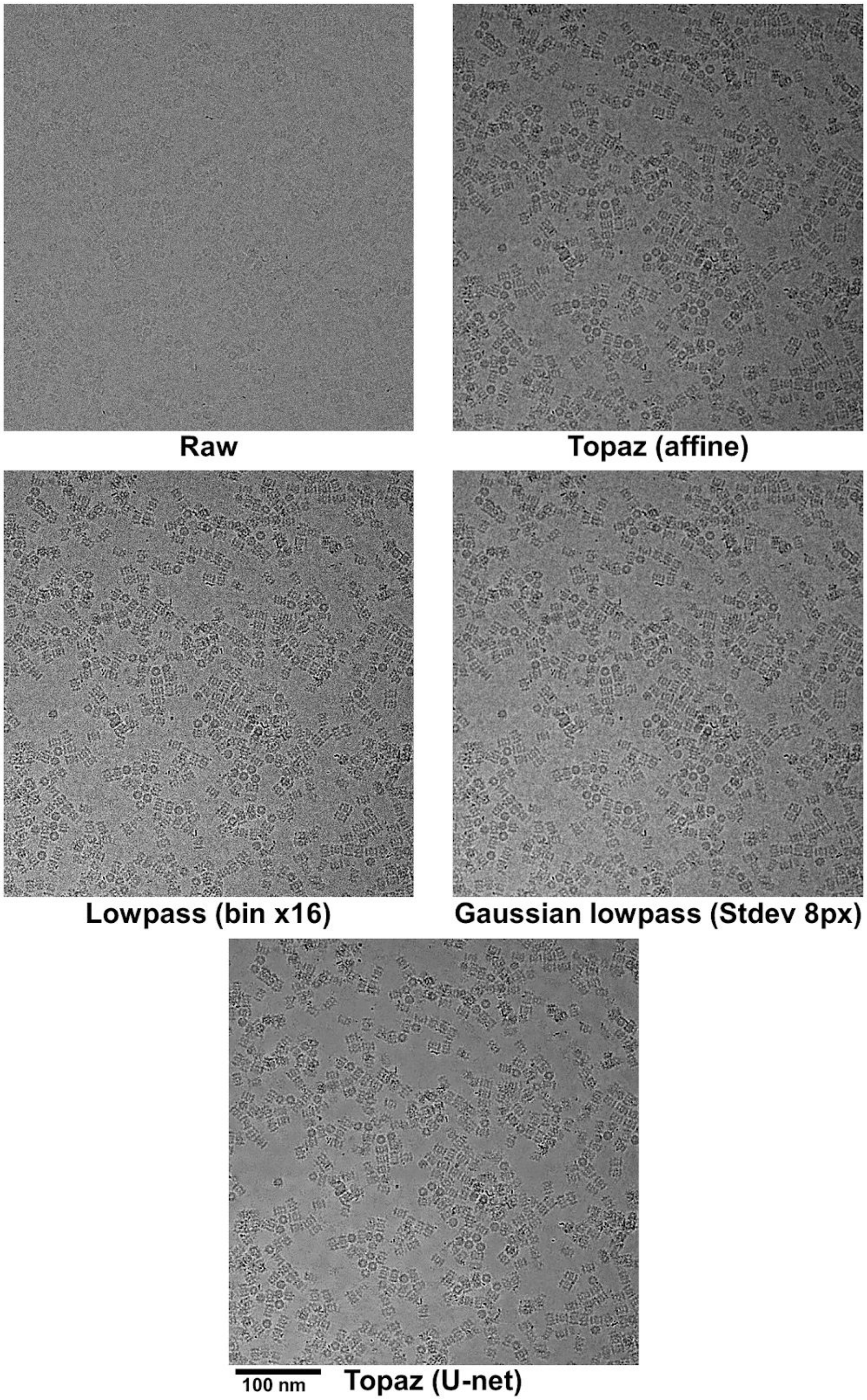

Supplementary Figure 1 | Comparison of denoising methods. Figure 1b micrograph (pixel size: 0.6575 Å) processed in four different ways: Topaz affine denoising model, low-pass binning by Fourier cropping by a factor of 16 then padding, Gaussian low-pass filtering with a standard deviation of 8 pixels, and our Topaz U-net denoising model.

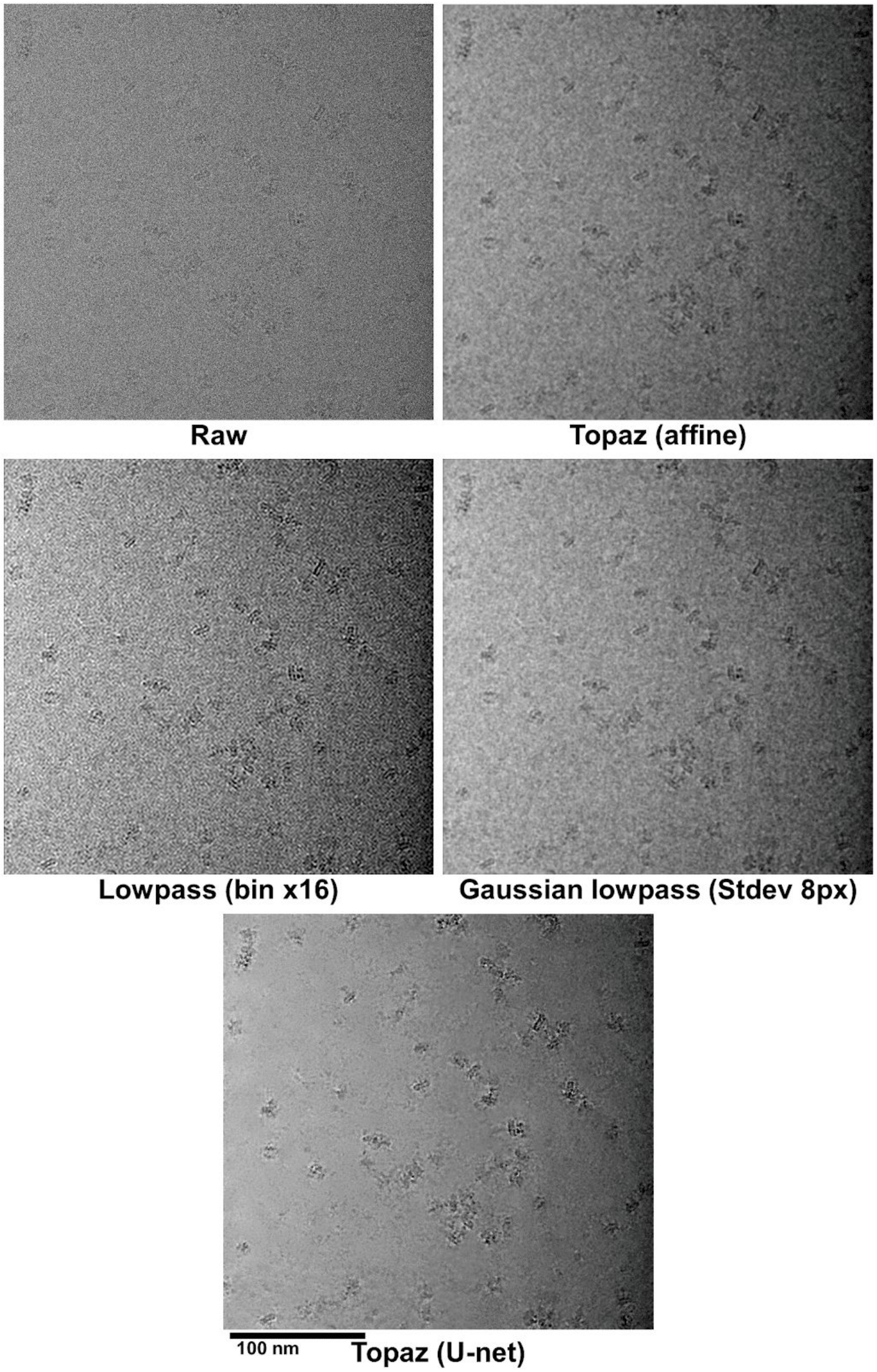

Supplementary Figure 2 | Comparison of denoising methods on EMPIAR-10261. Figure 1c micrograph (pixel size: 0.849 Å) processed in four different ways: Topaz affine denoising model, low-pass binning by Fourier cropping by a factor of 16 then padding, Gaussian low-pass filtering with a standard deviation of 8 pixels, and our Topaz U-net denoising model.

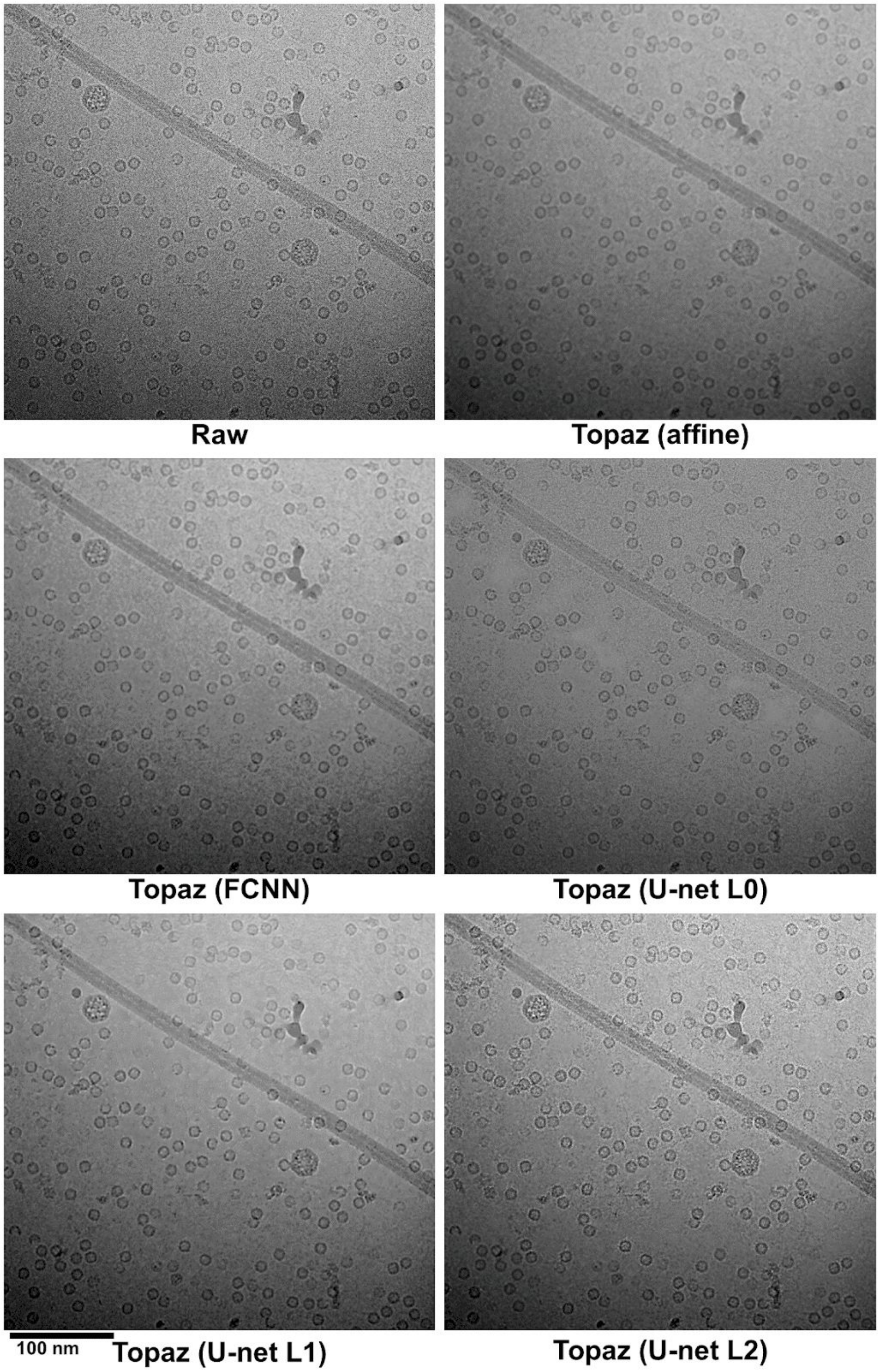

Supplementary Figure 3 | Comparison of Topaz neural denoising models on 19jan04d. Denoising models are compared on a micrograph (pixel size 1.10 Å) of apoferritin, β-galactosidase, VLPs, and TMV. The raw image denoised with the affine model, FCNN model, U-net with mode-seeking L0 loss, U-net with median-seeking L1 loss, and U-net with mean-seeking L2 loss are shown.

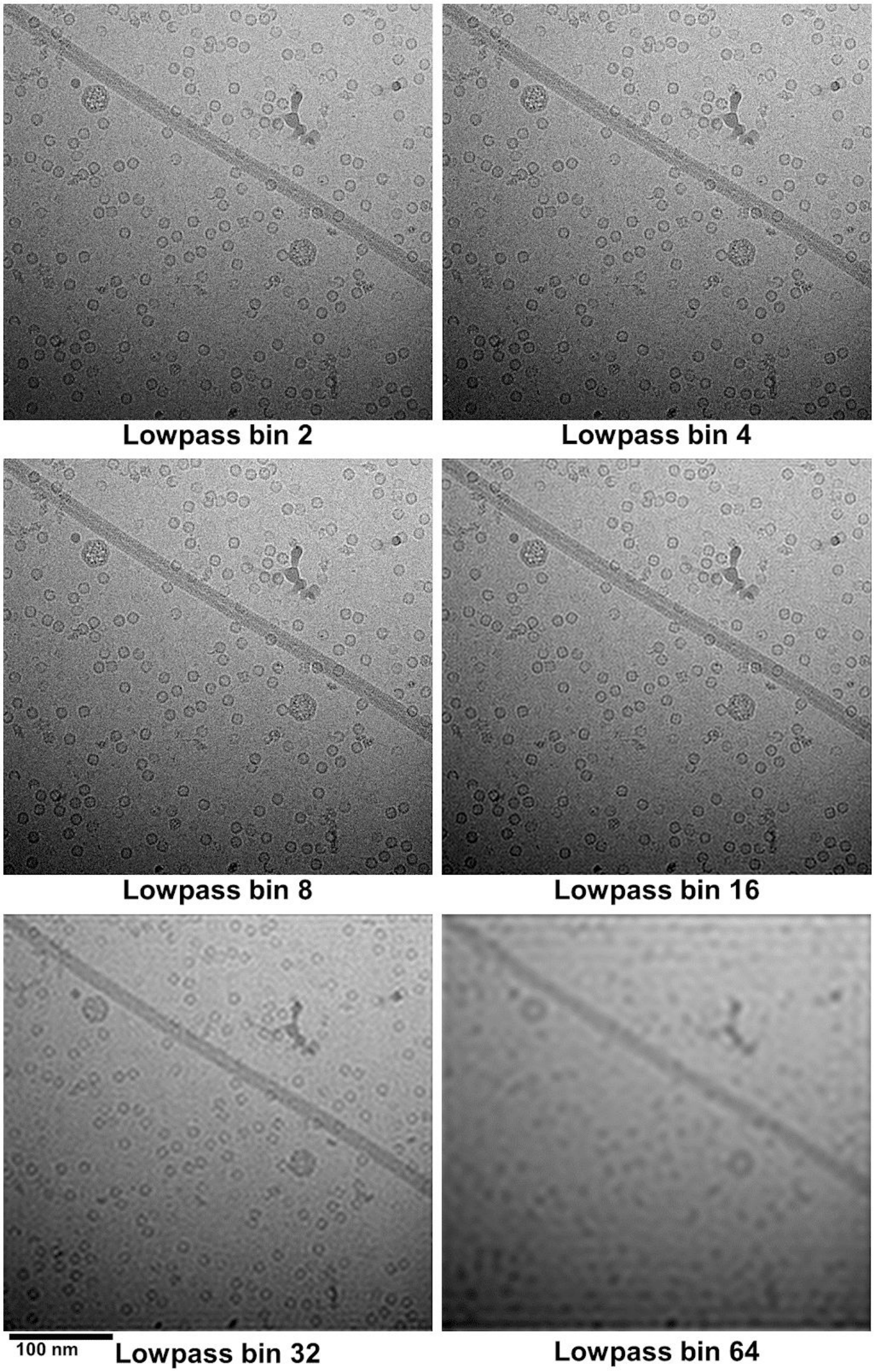

Supplementary Figure 4 | Comparison between low-pass binning by Fourier cropping on 19jan04d. The raw micrograph in Supplementary Figure 3 is low-pass filtered by factors of 2, 4, 8, 16, 32, and 64.

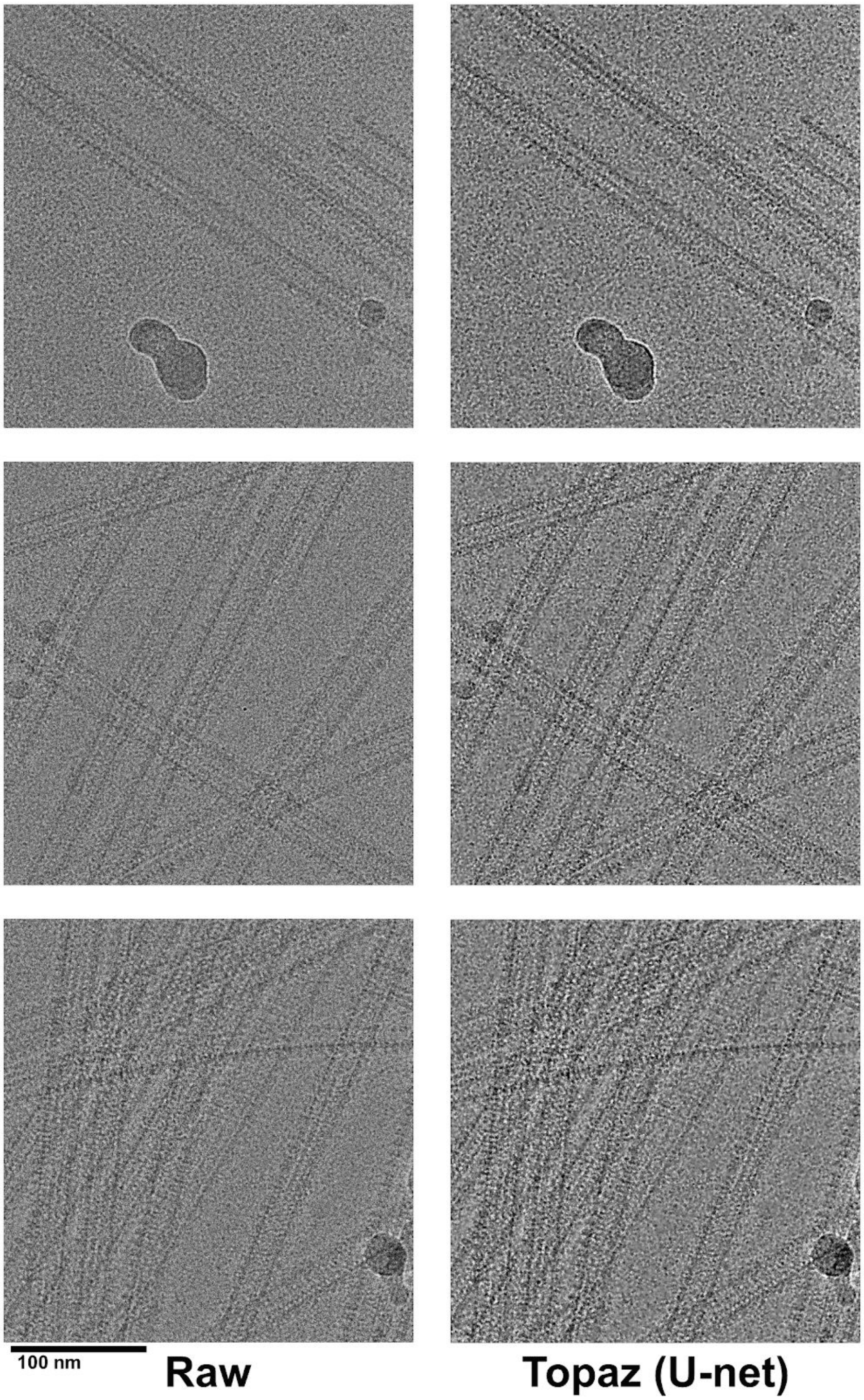

Supplementary Figure 5 | Denoising raw micrographs (18sep15a) of microtubules with known background contaminant proteins, kinesin and tubulin. Topaz denoising appropriately accentuates features of the background proteins instead of smoothing them out.

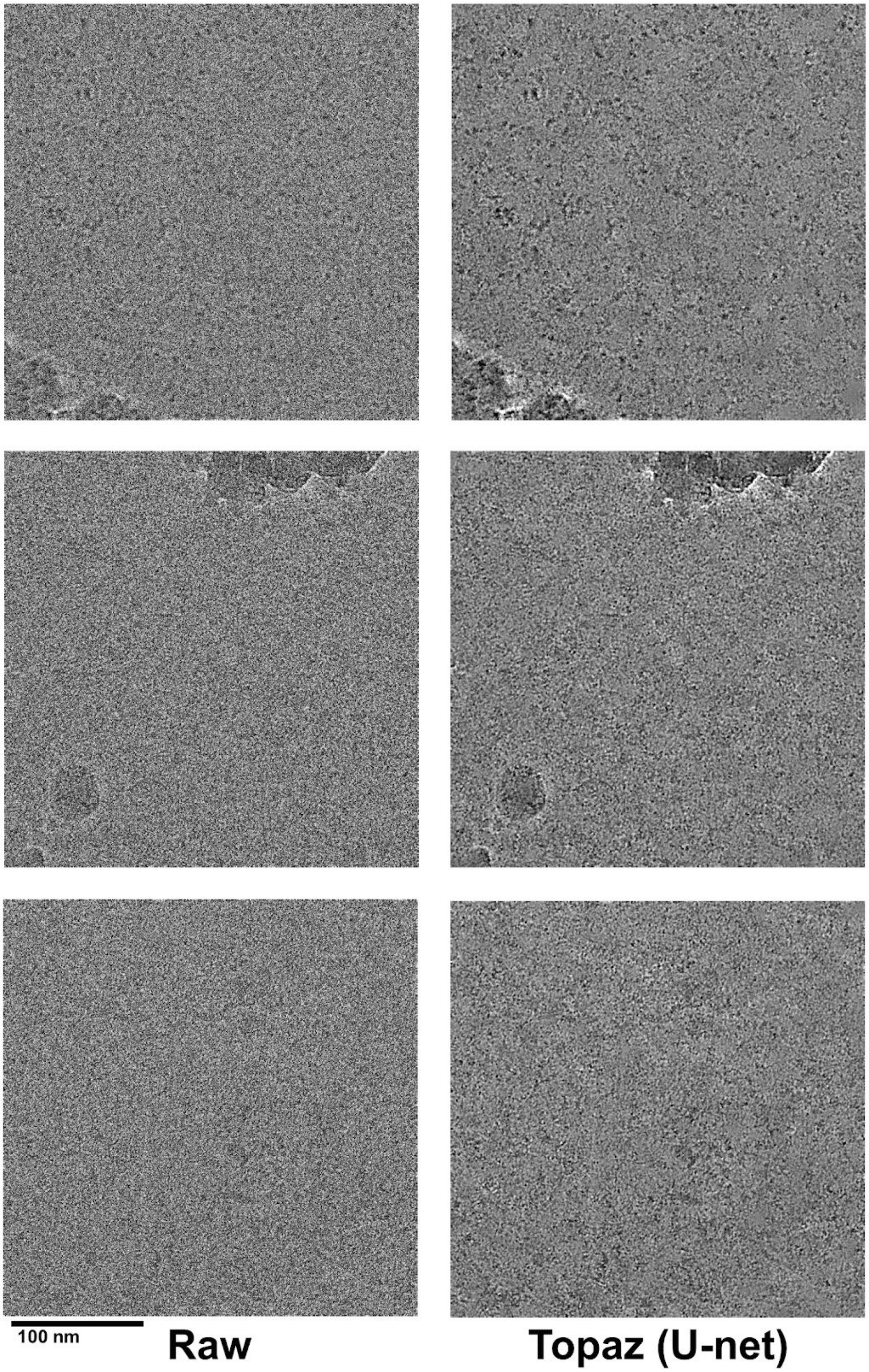

Supplementary Figure 6 | Denoising of EMPIAR-10003 raw images (left) using the U-net model (right). Possible regions of proteins are particularly apparent in the top image.

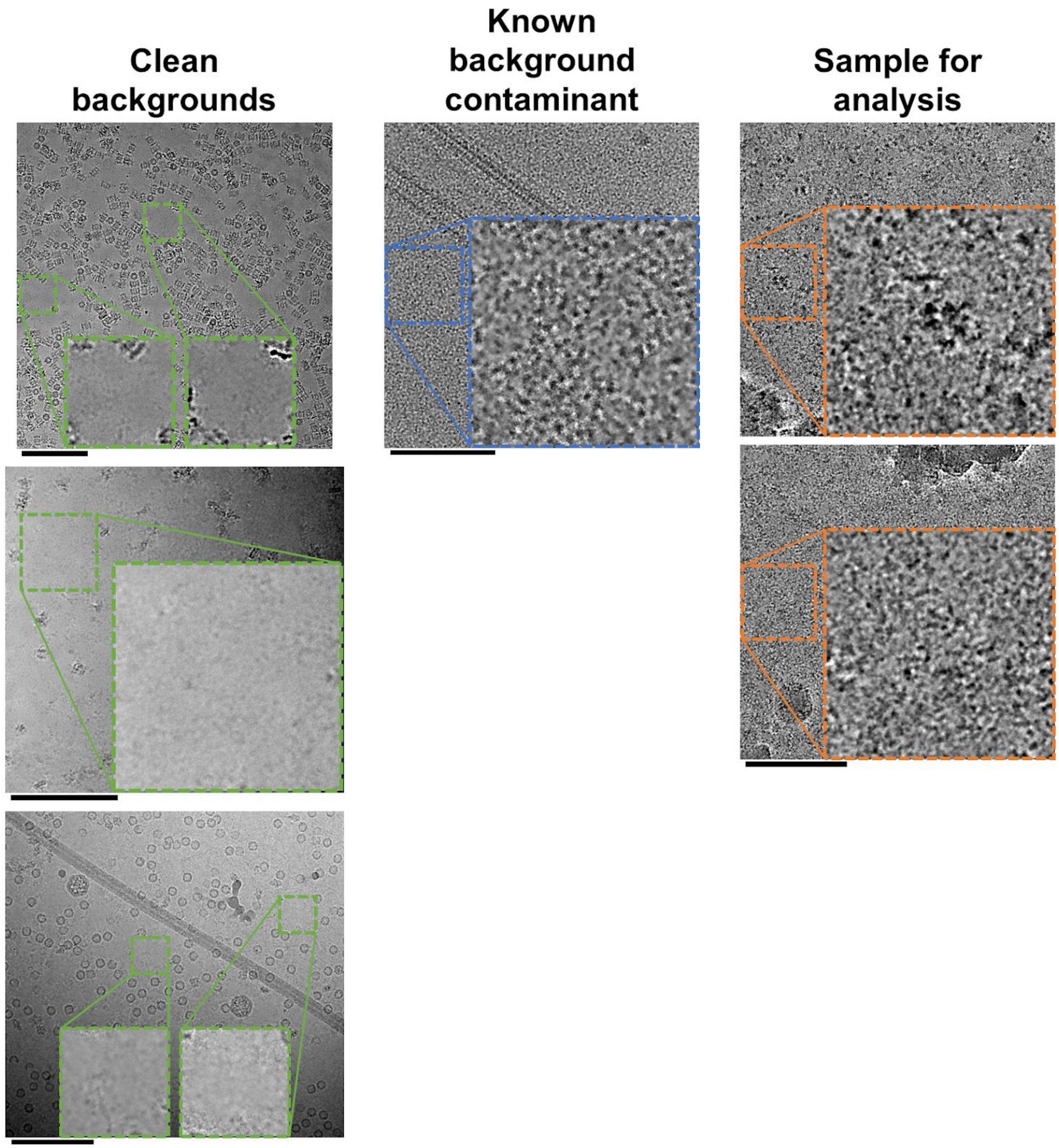

Supplementary Figure 7 | Denoising as a complementary method for analyzing background proteins and contamination in sample/grid preparations. Left: Three micrographs with nearly clean backgrounds (green insets). Middle: A micrograph of microtubules with known kinesin and tubulin background contaminant (blue inset). Right: Two micrographs from the EMPIAR-10003 dataset with the centers magnified (orange insets). All micrographs are denoised using our Topaz U-net model. Insets are magnified by 2x. Scalebars are 100nm.

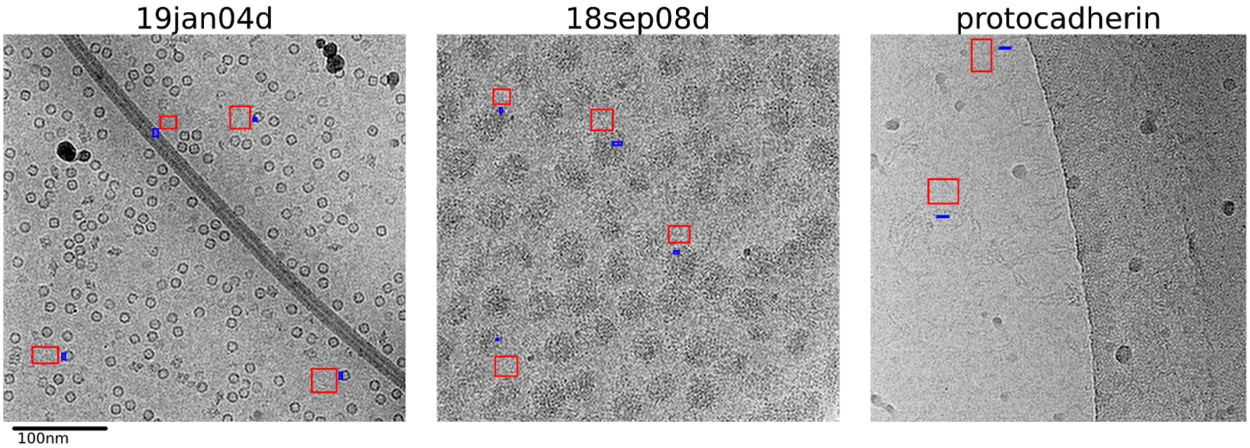

Supplementary Figure 8 | Paired signal and background for SNR quantification. Example micrographs from 19jan04d, 18sep08d, and the protocadherin dataset showing labeled signal (blue) and background (red) regions overlayed over low-pass filtered images. Signal and background regions were selected close together to match local background properties as best as possible to each signal region.

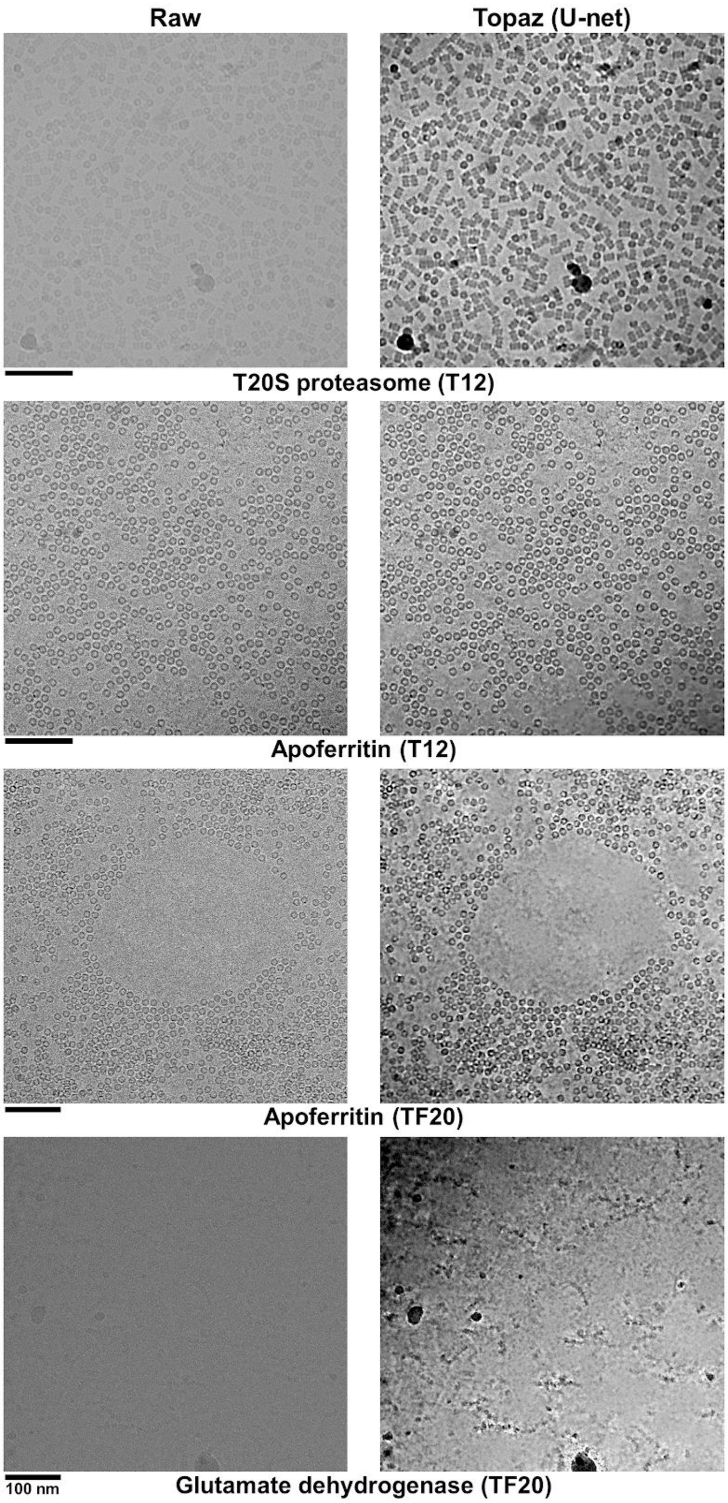

Supplementary Figure 9 | Denoising micrographs from screening microscopes. Hardware-binned-by-two micrographs from 120kV (T12) and 200kV (TF20) screening microscopes with TVIPS 4kTemCam-F416 scintillator-based cameras denoised with the general U-net model.

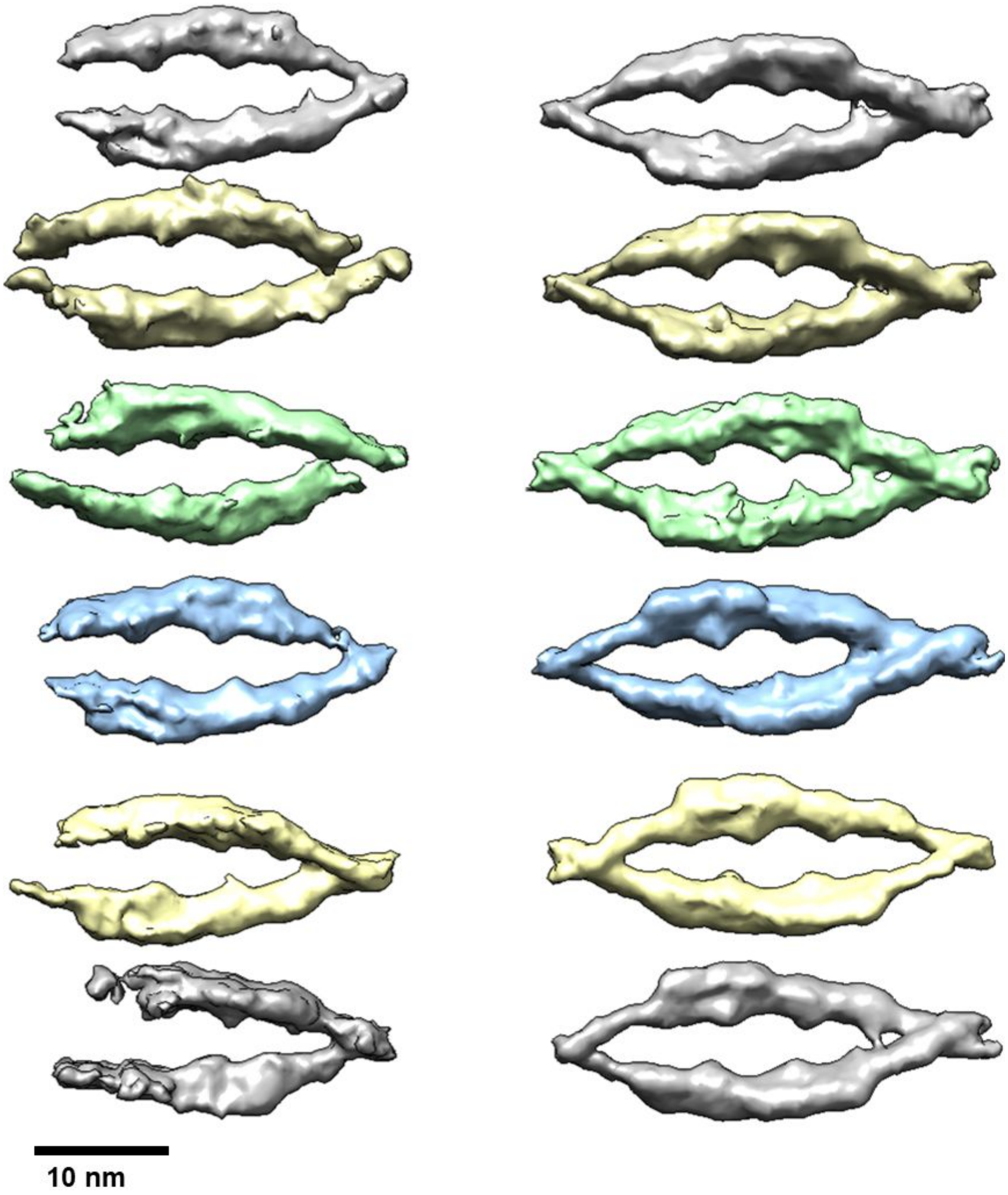

Supplementary Figure 10 | Reproducing ab-initio reconstruction with two classes.
CryoSPARC ab-initio reconstruction with two classes performed six times (rows) shows that the putative partially open state (left) and the closed state (right) are reproducibly generated.

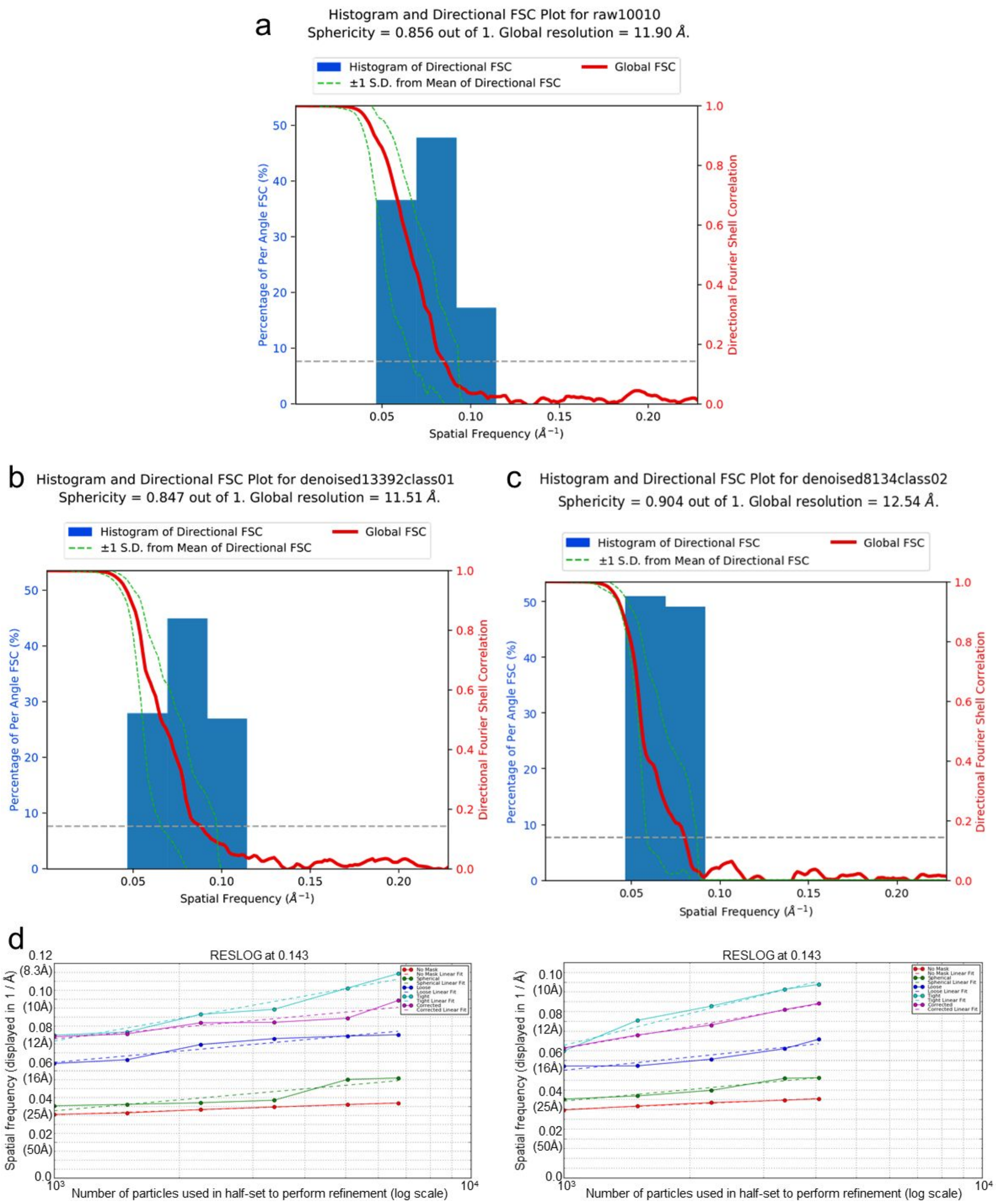

Supplementary Figure 11 | Denoising improves manual picking completion for difficult particles. 3DFSC and ResLog plots of clustered protocadherin (EMPIAR-10234). **(a)** Using the particle picks reported in Brasch et al., 2019, resulting in 10,010 particles in the 3D reconstruction (Figure 2b, left). **(b)** Using the particle picks reported on in the Methods (from manually picking on denoised micrographs prior to Topaz picking training on raw micrographs), resulting in 13,392 particles in the class 1 3D reconstruction (Figure 2b, middle) and **(c)** 8,134 particles in the class 2 3D reconstruction (Figure 2b, right). **(d)** ResLog plots of the closed state (left) and the putative partially open state (right).

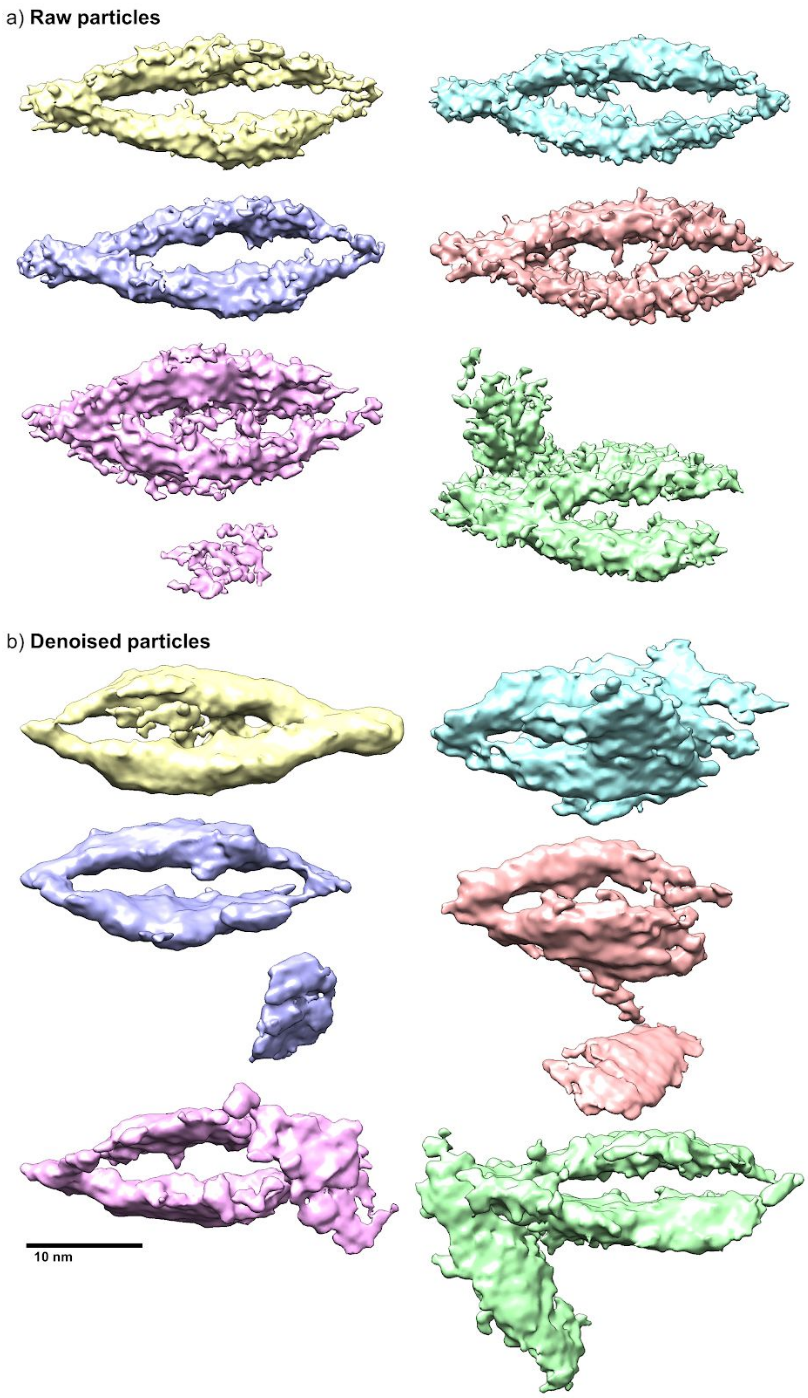

Supplementary Figure 12 | Denoised particles are less reliable for ab-initio model generation. 1,000 random particles processed through CryoSPARC ab-initio reconstruction using raw particles **(a)** and denoised particles **(b)**. 4 out of 6 reconstructions using raw particles result in the correct structure (a), while at best 1 out of 6 reconstructions using denoised particles result in the correct structure (b).

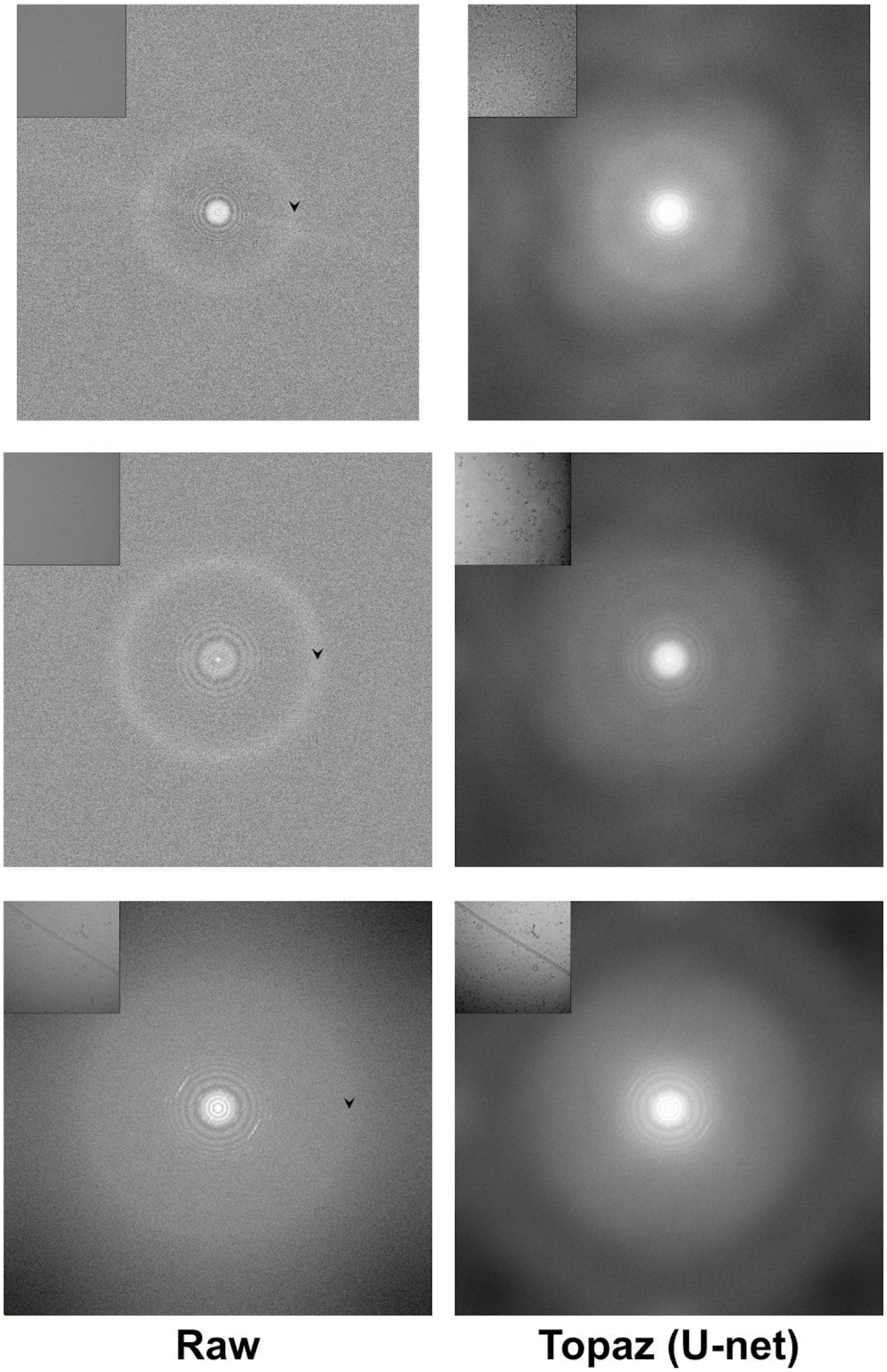

Supplementary Figure 13 | Fourier transforms of raw and denoised micrographs. Top: Figure 1b, EMPIAR-10025. Middle: Figure 3, EMPIAR-10261. Bottom: Figure 3, 19jan04d. Arrows show the location of the ~3.7 Å ice ring.

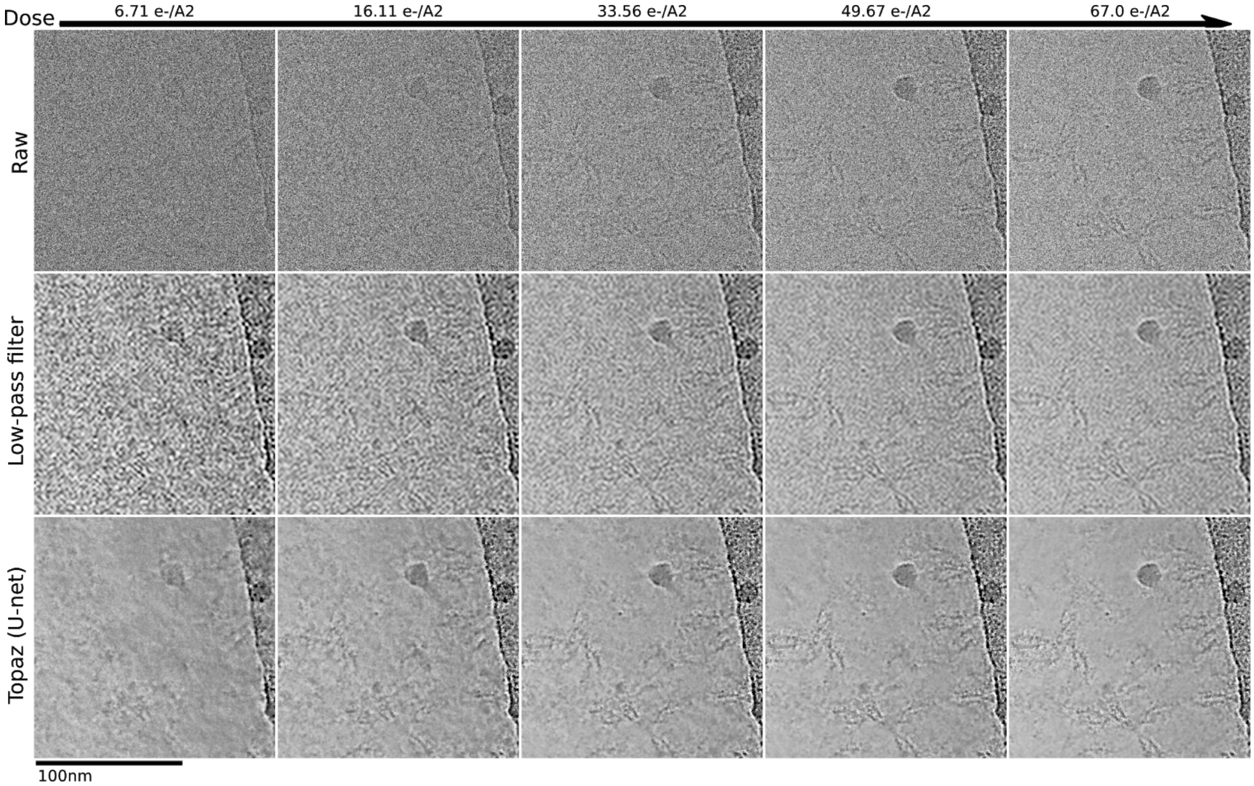

Supplementary Figure 14 | Short exposure detail of protocadherin. Detail of protocadherin micrograph denoised and raw over increasing dose.

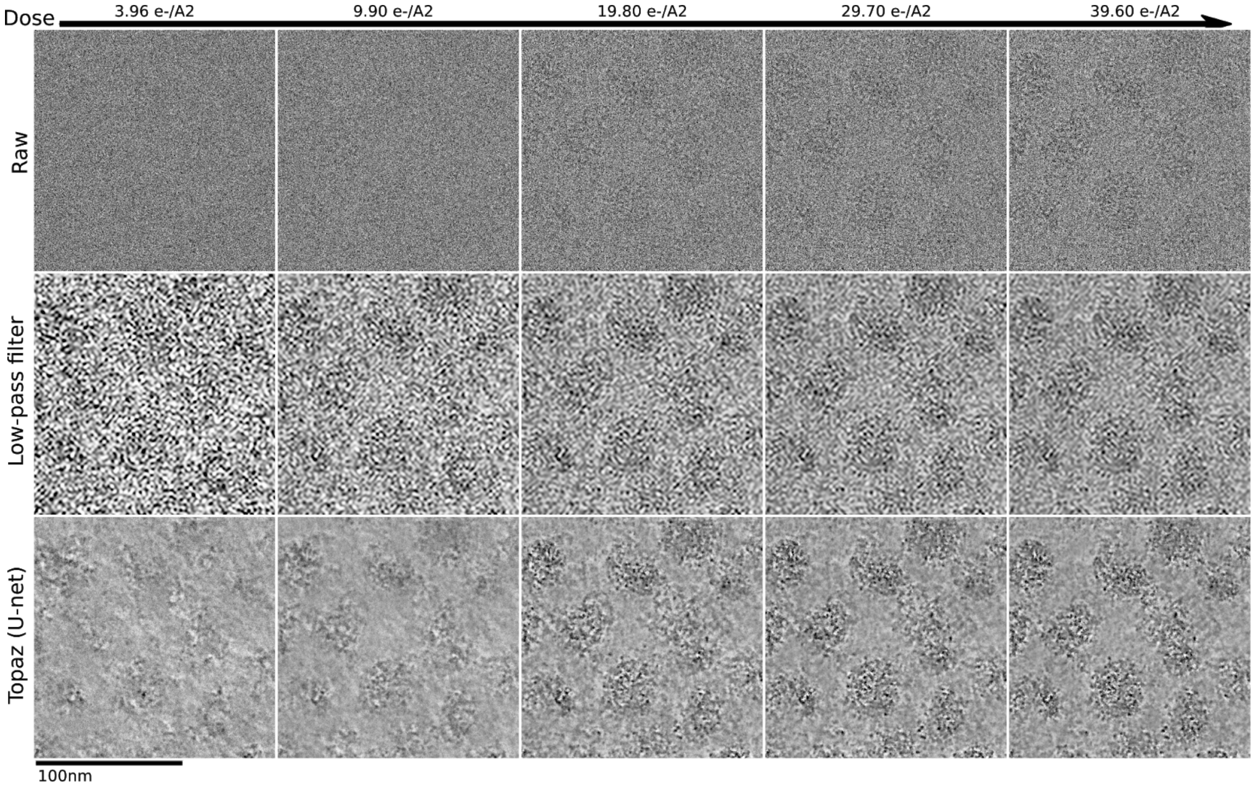

Supplementary Figure 15 | Short exposure detail of 18sep08d (VLPs). Detail of 18sep08d micrograph denoised and raw over increasing dose.

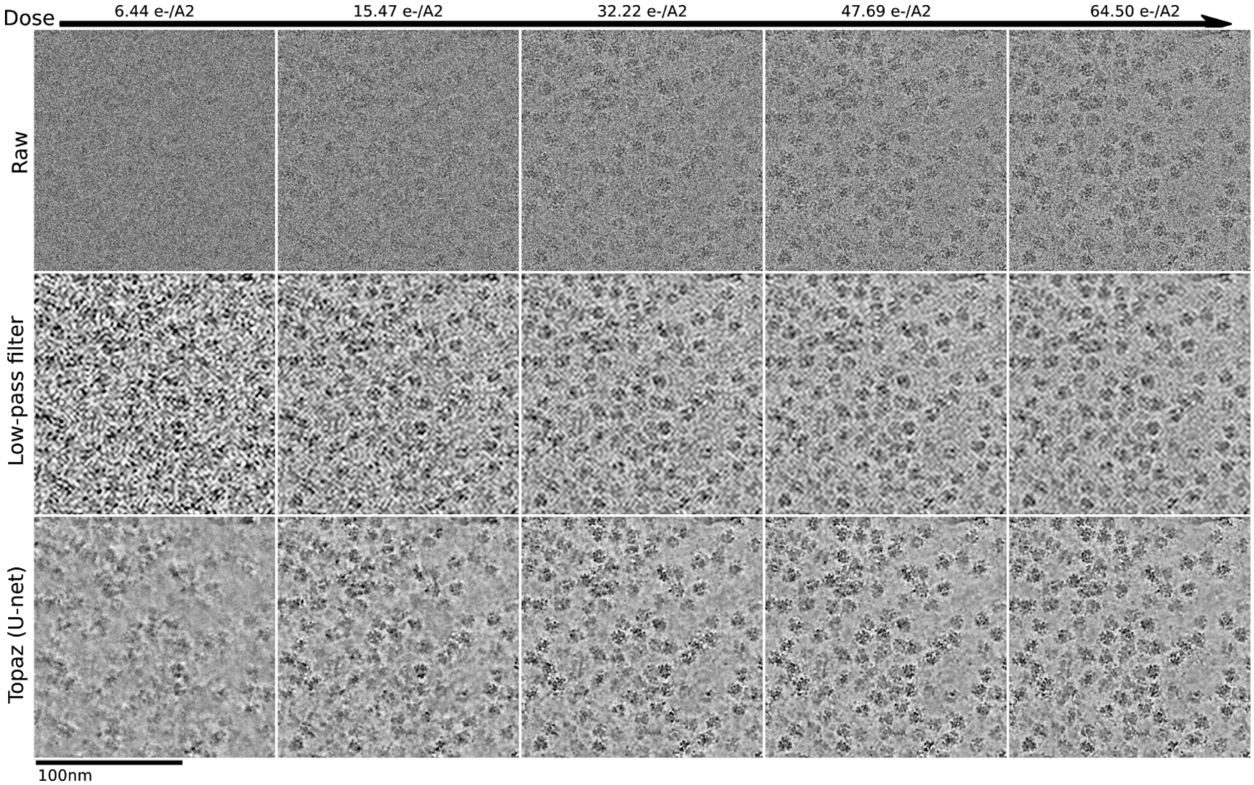

Supplementary Figure 16 | Short exposure detail of 19may10e. Detail of 19may10e micrograph denoised and raw over increasing dose.

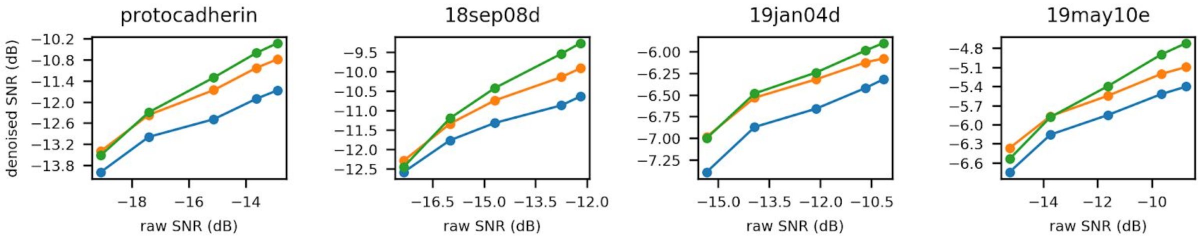

Supplementary Figure 17 | Estimated raw vs estimated denoised SNRs of short exposure micrographs. Plots of estimated raw SNR vs. estimated denoised SNR for short exposure micrographs accompanying Figure 3a. Micrographs were denoised with either a 16x low-pass filter (blue), the affine denoising model (orange), or our general purpose U-net model (green). SNRs were estimated using the split-frames method described in the Methods.

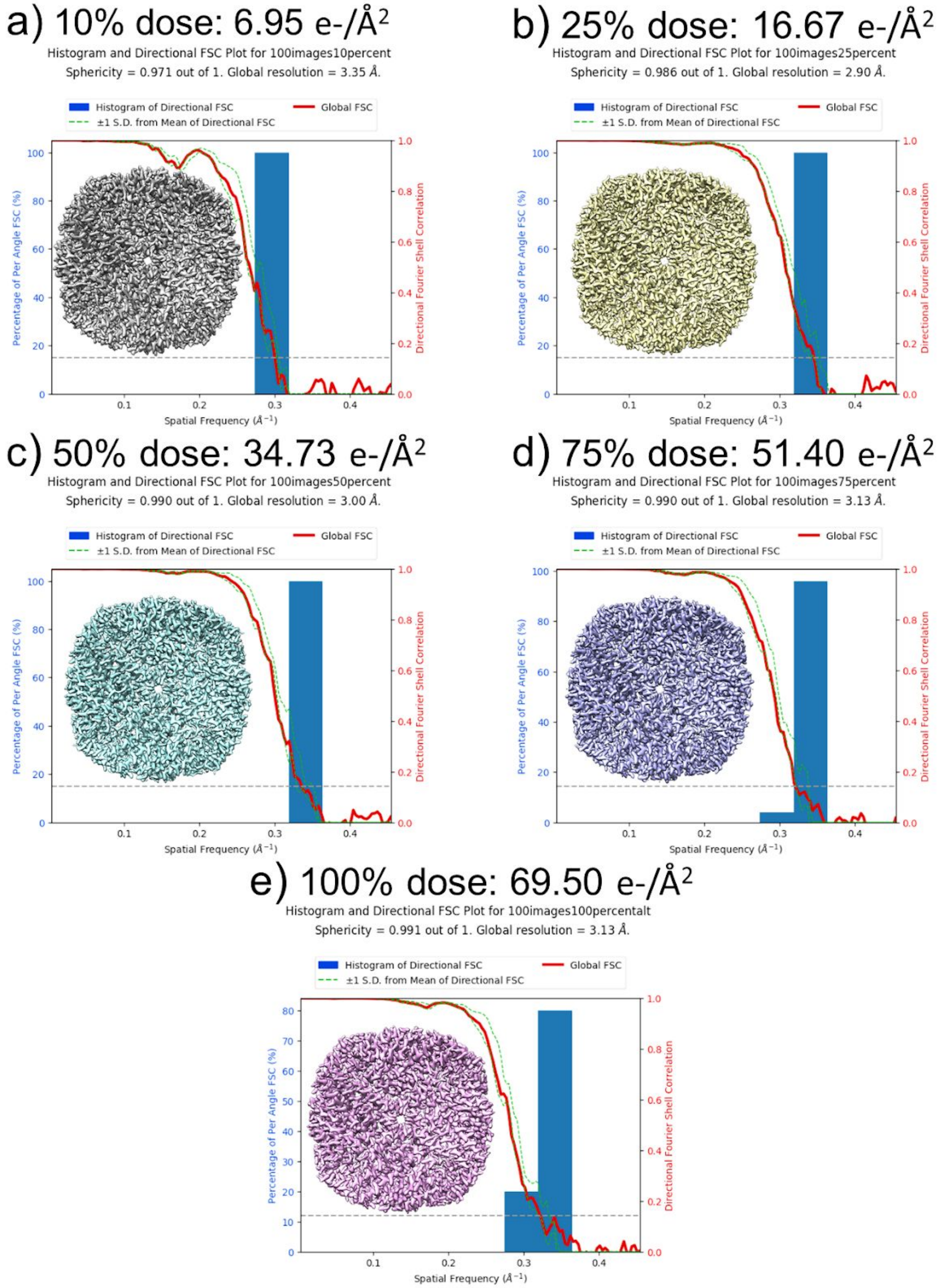

Supplementary Figure 18 | Short exposure 3D reconstructions of apoferritin. 3DFSC plots of apoferritin particles fractionated by frames/exposure time: **(a)** 6.95 e-/Å^2, **(b)** 16.67 e-/Å^2, **(c)** 34.73 e-/Å^2, **(d)** 51.40 e-/Å^2, and **(e)** 69.50 e-/Å^2.

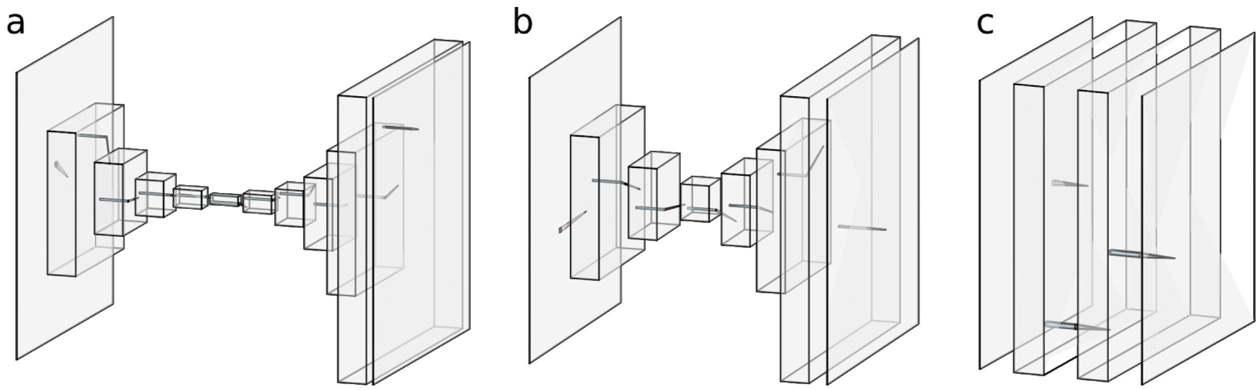

Supplementary Figure 19 | Model architectures. Model architecture diagrams for the U-net (a), small U-net (b), and FCNN (b) models. **(a)** The U-net model consists of 5 convolutional and downsampling blocks followed by 5 convolutional and upsampling blocks. Skip connections link each downsampling block to the mirrored upsampling block (not shown). Each convolutional layer has 48 filters. **(b)** The small U-net model has 3 convolutional and downsampling blocks followed by 3 convolutional and upsampling blocks. Skip connections link each downsampling block to the mirrored upsampling block (not shown). Each convolutional layer has 48 filters. **(c)** The FCNN model has three convolutional layers each of width 11 and 64 filters.

Supplementary References

50. Yu, Q., Qu, K. & Modis, Y. Cryo-EM structures of MDA5-dsRNA filaments at different stages of ATP hydrolysis. *Mol. Cell* **72**, 999–1012.e6 (2018).

51. Zhang, W. et al. Heparin-induced tau filaments are polymorphic and differ from those in Alzheimer's and Pick's diseases. *eLife* **8**, e43584 (2019).

52. Kujirai, T. et al. Structural basis of the nucleosome transition during RNA polymerase II passage. *Science* **362**, 595–598 (2018).

53. Kato, T. et al. EMPIAR-10248 dataset: The 1.54 Å structure of Apoferritin by CRYOARM300 with cold-FEG. https://doi.org/10.6019/EMPIAR-10248 (2019).

54. Laughlin, T. G., Bayne, A. N., Trempe, J.-F., Savage, D. F. & Davies, K. M. Structure of the complex I-like molecule NDH of oxygenic photosynthesis. *Nature* **566**, 411–414 (2019).

55. Kern, D. M., Oh, S., Hite, R. K. & Brohawn, S. G. Cryo-EM structures of the DCPIB-inhibited volume-regulated anion channel LRRC8A in lipid nanodiscs. *eLife* **8**, e42636 (2019).

56. Xu, H. et al. Correction: structural basis for the prion-like MAVS filaments in antiviral innate immunity. *eLife* **4**, e07546 (2015).

57. Bartesaghi, A. et al. 2.2 Å resolution cryo-EM structure of β-galactosidase in complex with a cell-permeant inhibitor. *Science* **348**, 1147–1151 (2015).

58. Wong, W. et al. Cryo-EM structure of the Plasmodium falciparum 80S ribosome bound to the anti-protozoan drug emetine. *eLife* **3**, e03080 (2014).
