# Supplementary material for: Topaz-Denoise: general deep denoising models for cryoEM and cryoET
Source: Nat Commun. 2020 Oct 15;11:5208. doi: 10.1038/s41467-020-18952-1 (PMC7567117; doi:10.1038/s41467-020-18952-1)
Supplement: Supplementary file 3 — Description of Additional Supplementary Files [file 41467_2020_18952_MOESM3_ESM.pdf]

Description of Additional Supplementary Files

File Name: Supplementary Movie 1
Description: 3D denoising comparison of Saccharomyces uvarum lamellae. Visual comparison of cryoET weighted back-projection tomogram slice-throughs of the yeast lamellae in Figure 4a. The tilt images were binned by 2 prior to reconstruction. Top-left: The original tomogram without filters. Top-right: The original tomogram binned by another factor of 4 in Fourier space (total binning of 8). Bottom-left: The original tomogram denoised with a 3D denoising model trained on even/odd halves of the same tomogram. Bottom-right: The original tomogram denoised with Unet-3d-10a.

File Name: Supplementary Movie 2
Description: 3D denoising comparison of 80S ribosomes from EMPIAR-10045. Visual comparison of cryoET weighted back-projection tomogram slice-throughs of the single particle ribosomes in Figure 4b. Left: The original tomogram without filters or binning. Middle: The original tomogram binned by a factor of 8 in Fourier space. Right: The original tomogram denoised with Unet-3d-10a.
